# Genetic Variation in Bank Vole Populations in Natural and Metal-Contaminated Areas

**DOI:** 10.1007/s00244-014-0036-4

**Published:** 2014-05-20

**Authors:** Magdalena Mikowska, Aneta Gaura, Edyta Sadowska, Paweł Koteja, Renata Świergosz-Kowalewska

**Affiliations:** Institute of Environmental Sciences, Jagiellonian University, Gronostajowa 7, 30-387 Krakow, Poland

## Abstract

The effects of isolation and heavy-metal pollution on genetic diversity in *Myodes* (=*Clethrionomys*) *glareolus* populations were studied. Isolation and pollution are considered to have important effects on biodiversity. Animals were collected from ten populations in isolated (island), mainland, and metal-polluted areas. Three populations were in areas near zinc and lead smelters; four were on islands in the relatively unpolluted Mazurian Lake District and in the Bieszczady Mountains; and three were in clean-mainland areas in the Mazurian Lake District, the Niepołomice Forest, and the Bieszczady Mountains. Cadmium and lead concentrations in liver and kidney were measured to assess the animals’ exposure to metals. The metal concentrations were greater in animals from areas classed as polluted than in animals from the clean-mainland areas and islands. The genetic diversity of each population was analyzed using eight microsatellite markers. The results confirmed that isolation adversely affects genetic diversity in *M. glareolus* populations (giving low heterozygosity and poor allelic richness), but the effect of metal exposure on genetic diversity was not strong. Of the samples from polluted areas, only the Katowice population, which is exposed to high levels of metal pollution and is also isolated because of human activity, showed genetic variation parameters that were similar to those for the island populations. Nei’s genetic distances indicated that the island populations were genetically distant from each other and from the other populations, and there were noticeable inbreeding effects that would have been caused by the isolation of these populations.

Ecological and ecotoxicological studies are being increasingly focused on diversity loss, which has been studied during the last few decades at various organization levels: genetic diversity in a single species, species richness in communities, and biodiversity in ecosystems (Booy et al. 2000; Franklin 1993). The main concern in conservation ecology is still genetic diversity in endangered and “keystone” species (the loss of which can disturb the stability of an ecosystem). However, genetic diversity is important in general, and it is crucial to determine the processes and factors responsible for the loss of genetic diversity in common species. The genetic variation in a number of common species is still poorly known, especially in terms of its relationship with isolation and exposure to environmental pollution. Because of this, we chose a common rodent, the bank vole, for the study presented here.

Genetic variation is the basis of evolution, allowing organisms to adapt to changing environmental conditions (Hartl and Clark 2010). The main sources of variation are point mutation and genetic recombination (Charon and Świtoński 2004). Many factors that affect organisms (natural and anthropogenic, chemical, and physical) can increase or decrease genetic variation depending on the characteristics of the acting factor. Migration, hybridization, and point mutation are known to increase genetic variation, but selection by disease or climate change, which will decrease survival rates, can cause a bottleneck effect and decrease genetic diversity (Bickham et al. 2000).

Some of the factors mentioned previously may be examples of anthropogenic stress, which is thought to cause genetic erosion (Van Straalen and Timmermans 2002). Ribeiro and Lopes (2013) described genetic erosion as a “loss of genotypes determining a specific trait or set of traits.” Van Straalen and Timmermans (2002) specified four processes that may contribute to genetic erosion: increased mutation rates, directional selection, the bottleneck effect, and disturbed migration. However, the theory is supported by the results of a number of published studies and contradicted by the results of others. For example, no correlation was found between genetic diversity and metal pollution in the wood mouse (*Apodemus sylvaticus*) by Berckmoes et al. (2005), and no correlation was found between metal exposure and the genetic variance within populations of *Porcellionides saxfasciatus* by Costa et al. (2013). However, Berckmoes et al. (2005) found contamination-related patterns in the genetic structures of wood mice at different sites. Van Straalen and Timmermans (2002) stated that there is support from various studies for the genetic erosion theory but also that “the issue cannot be considered settled.” The theory may also be supported by recently published data for *Peromyscus melanophrys* (an endemic small mammal in Mexico) showing a significant negative relationship between genetic diversity and metal concentration (Mussali-Galante et al. 2013). The impact of pollution on genetic diversity in a population is well documented for radionuclides (Theodorakis et al. 2001; Theodorakis and Shugart 1997), but the effects of other types of toxic substances have not been fully explained, and research in this area is continuing (Costa et al. 2013; Ellegren et al. 1997; Fratini et al. 2008; Mussali-Galante et al. 2013). The results of previous studies have suggested that indirect negative effects of pollutants (through decreased survival or reproductive success) cannot be excluded, especially in small populations (Berckmoes et al. 2005). Understanding the early causes of diversity loss could help us to prevent processes such as bottlenecks and extinction. We need such data on species that are used in ecotoxicological research to better understand and interpret the results of other field works.

The main goal of our investigation was to examine genetic variations in bank vole populations and to determine whether natural factors (isolation on an island in a lake) and environmental factors (exposure to metal pollution) affect these variations. Isolation by natural barriers (e.g., water), artificial barriers (e.g., roads and industrial infrastructure), and geographic distance have been shown to be important factors in the loss of diversity. Geographic distance sometimes has a less significant effect than do other barriers, such as isolation by a stretch of water. Kozakiewicz et al. (2009) showed that the intensity of this effect depends on the species, and they found that the “isolation effect” on islands is more profound for bank vole populations than for yellow-necked mouse populations. The investigators also concluded that these differences are not caused by differences in the mobility of the two species but rather by differences in other aspects of the behaviors of the species. Hinten et al. (2003) found low levels of genetic variation in isolated island populations of the Australian bush rat *Rattus fuscipes greyii*, high levels of genetic variation between populations, and less diversity in island populations than in the mainland populations. Industrialization has been found to pose threats from both pollution and habitat fragmentation in some regions. We used the information described previously as the basis for choosing the species to use as a subject in the investigation presented here and for identifying suitable study sites.

The bank vole, *M. glareolus*, chosen for the study, is one of many common species that have been used as model species in research into universal physiological processes in organisms in threatened environments. The main reason for choosing this species is that it is very common and that it inhabits a variety of environments, including areas close to industrial regions (Pucek 1984). The genetic constitution of the bank vole is not well studied, and information on this would be useful for interpreting other ecotoxicological data. We used microsatellites, as appropriate, and previously tested markers to achieve our goals. These codominant markers have been successfully used to identify individuals and to assess genetic variations in populations of different species (Kozakiewicz et al. 2009; Łagisz et al. 2010).

Our hypotheses were: (1) animals from areas near zinc/lead smelters have greater metal concentrations in their tissues than animals from other populations; (2) populations on islands have lower levels of genetic diversity than do populations in clean and open site, because of the isolation effect; and (3) populations in polluted areas have low levels of genetic diversity because of the direct and indirect effects of metal pollution. We selected three types of bank vole populations in southern and northeastern Poland. The populations inhabit chronically polluted sites, clean-mainland areas, and islands in lakes.

## Materials and Methods

### Trapping and Study Sites

We collected 197 *M. glareolus* individuals (both sexes) from ten study sites between August and November 2009 using a standard live-trapping technique. The trapped animals were transported to the laboratory in plastic cages and then killed by decapitation. Kidney and liver tissues were collected and stored at −75 °C until they were subjected to chemical analysis. Part of the auricle from each individual was preserved in 96 % ethyl alcohol solution (POCH S.A., Gliwice, Poland) and stored at −75 °C, for molecular analysis. All procedures were performed according to EC Directive 86/609/EEC for animal experiments.

**Fig. 1:**
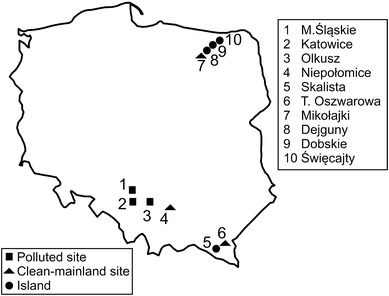
Map of Poland indicating study areas. Geometric figures represent different population types. *Numbers* represent each study site/population

The collection sites were in northeastern and southern Poland, and they are shown in Fig. 1. The populations are identified by an abbreviation, the first letter of which indicates the type of population (I = island, P = polluted area, C = clean-mainland area) followed by a site identifier.

Three of the island populations that were sampled were in the Mazurian Lake District in northern Poland (labelled North), and one was in the Bieszczady Mountains in southern Poland (labelled South). The three North islands do not have geographical names, so we labeled them with the names of the lakes they are in:

- Dobskie (IDo)—54°5′N, 21°36′E, area 1.5 ha, approximately 500 m from the shore, North;
- Dejguny (IDe)—54°2′N, 21°37′E, area 9.3 ha, approximately 250 m from the shore, North;
- Święcajty (ISw)—54°11′N, 21°45′E, area 7.4 ha, approximately 400 m from the shore, North;
- Skalista (ISo)—49°21′N, 22°30′E, area 3.0 ha, approximately 250 m from the shore, South.

The three polluted sites were close to zinc and lead smelters in Silesia Province (southern Poland), and the environment in each area was contaminated with a similar combination of pollutants. The polluted sites were as follows:

- Olkusz (POl)—50°19′N, 19°30′E;
- Miasteczko Śląskie (PMi)—50°31′N, 18°55′E;
- Katowice (PKa)—50°15′N, 19°4′E.

We were unable to find a set of similarly polluted areas in northern Poland. The POl site is near the Bolesław zinc/lead mine and smelter, and high concentrations of contaminants have been reported in this area for several decades (Verner et al. 1996). The PKa site is near the recently closed Szopienice zinc/lead smelter, which was a major source of emissions of zinc, lead, and cadmium compounds. The PMi smelter is the largest production plant of zinc and lead in Poland. The high concentration of mines and smelters in Silesia Province has led to high concentrations of a range of metals being present in the soils (Ullrich et al. 1999; Verner et al. 1996) and mosses (Grodzińska et al. 2003) in the region. Lead concentrations of 2,046 mg/kg have been found in soils from Żyglinek near PMi (Syrek et al. 2006). Lead concentrations of 870 mg/kg have been found in humus layer samples from Hutki near the Bolesław smelter (Łaszczyca et al. 2004). Zinc concentrations in soil samples from the Szopienice area have been found to be >10 times the permitted concentration (Augustyniak et al. 2008). Metal contamination in these areas has been described in detail by Augustyniak and Migula (2000), Augustyniak et al. (2005), Łaszczyca et al. (2004), Stone et al. (2001), and Syrek et al. (2006).

The clean-mainland populations were near these areas:

- Mikołajki (CMi)—53°46′N, 21°30′E, Mazurian Lake District, North, approximately 30 km from the closest of the island populations that were studied);
- Niepołomice (CNi)—50°0′N, 20°20′E, South, approximately 20 km from Kraków);
- Teleśnica Oszwarowa (CTe)—49°22′N, 22°32′E, Bieszczady Mountains, South, 2 km from ISo Island).

### Chemical Analysis

Samples from a total of 115 individuals were collected for metal analyses (Table 1). Kidney and liver samples were dried to constant weight at 70 °C and then wet-digested in nitric acid (Suprapur; Merck, Darmstadt, Germany). The metal concentrations were determined by graphite furnace atomic absorption spectrometry (AAnalyst 800; Perkin-Elmer, Waltham, Massachusetts, USA). The concentrations are presented in units of milligrams of metal per kilogram of dry tissue. A certified reference material (SRM 1577c Bovine Liver; National Institute of Standards and Technology, Gaithersburg, Maryland, USA) was used to assess the analytical precision. The concentrations determined in the reference material differed from the certified concentrations (97.0 ± 1.4 µg/kg dry weight (dw) for Cd and 62.8 ± 1.0 µg/kg dw for Pb) by no more than 5 %. Chemical analysis was performed on samples from all of the populations except for the ISw population, from which too few individuals were caught.

**Table 1:** Cadmium and lead concentrations (mg/kg dw) in the kidney and liver of bank voles *M. glareolus* from various sites

Population (*N*)	Kidney				Liver			
	Cadmium (mg/kg dw)		Lead (mg/kg dw)		Cadmium (mg/kg dw)		Lead (mg/kg dw)	
	Mean ± SE*	Range	Mean ± SE	Range	Mean ± SE	Range	Mean ± SE	Range
Clean-mainland								
Mikołajki (CMi) (16)	0.94 ± 4.31^a^	0.21–3.45	0.12 ± 0.11^a^	0.00–0.70	0.33 ± 1.12^a^	0.04–1.42	0.19 ± 0.13^a^	0.07–0.40
Niepołomice (CNi) (11)	0.78 ± 5.20^a^	0.48–2.23	0.47 ± 0.13^ab^	0.02–1.94	0.38 ± 1.29^a^	0.08–2.43	0.23 ± 0.15^ab^	0.08–0.40
T. Oszwarowa (CTe) (12)	2.34 ± 4.97^ab^	0.41–12.60	0.24 ± 0.12^ab^	0.00–0.58	0.67 ± 1.29^a^	0.22–2.31	0.24 ± 0.15^ab^	0.05–0.67
Polluted								
Katowice (PKa) (11)	40.67 ± 5.20^c^	0.00–159.77	0.74 ± 0.13^bd^	0.30–1.52	10.44 ± 1.35^c^	0.23–31.45	0.23 ± 0.15^ab^	0.13–0.37
M. Śląskie (PMi) (10)	20.31 ± 5.74^c^	0.00–35.08	1.26 ± 0.14 ^cd^	0.79–2.21	6.17 ± 1.42^bc^	1.59–23.66	0.36 ± 0.16^ab^	0.15–0.54
Olkusz (POl) (14)	16.02 ± 4.61^c^	4.66–39.95	1.32 ± 0.11^c^	0.41–2.25	5.11 ± 1.20^b^	0.92–29.04	0.78 ± 0.13^b^	0.05–5.08
Island								
Dejguny (IDe) (13)	0.94 ± 4.78^a^	0.10–3.67	0.26 ± 0.12^ab^	0.00–0.66	0.23 ± 1.24^a^	0.06–0.65	0.21 ± 0.14^ab^	0.09–0.69
Dobskie (IDo) (16)	1.71 ± 4.31^ab^	0.16–8.48	0.44 ± 0.11^ab^	0.07–1.93	0.41 ± 1.12^a^	0.17–1.28	0.18 ± 0.13^a^	0.08–0.34
Skalista (ISo) (12)	6.04 ± 5.20^b^	0.68–12.50	0.37 ± 0.13^ab^	0.00–2.20	2.07 ± 1.29^ab^	0.23–4.82	0.30 ± 0.15^ab^	0.07–1.99

*Mean* arithmetic mean, *range* minimum and maximum concentration

* Values with different letters within columns indicate a significant difference between populations (*p* < 0.05)

### Genetic Analysis

DNA was isolated from an auricle fragment from each of the individuals that had been caught (*N* = 197) using the DNeasy blood and tissue kit (Qiagen, Hilden, Germany) using a protocol designed for animal tissue. Lysis was performed at 55 °C in a Thermomixer (Eppendorf, Hamburg, Germany). The DNA concentration was measured using a Nanodrop ND-1000 spectrophotometer (PEQLAB Biotechnologie GmbH, Erlangen, Germany).

The genetic diversity was assessed using eight polymorphic loci: MSCg-4, MSCg-9 (Gockel et al. 1997), MSCg-7, MSCg-24, MSCg-20 (Gerlach and Musolf 2000), LIST3-003, LIST3-005, and LIST3-002 (Barker et al. 2005). Polymerase chain reaction (PCR) with a temperature gradient was performed to find the best temperature for each locus. Two multiplexes were set up and run using Qiagen Multiplex PCR Kit using the optimized conditions, and, according to the protocol, each multiplex contained 25 µL of the master mix, 5 µL of the primer mix (the final concentration of each primer was 0.2 µM), 19 µL of RNase-free water, and 1 µL of DNA. The final concentration of MgCl_2_ was 3 mM. The vendor did not provide information on the amount of polymerase in the master mix. If the DNA concentration in the sample was <30 ng/µL, the mixture described previously was changed to include the following: 18 µL of RNase-free water and 2 µL of DNA, to avoid too low of a DNA concentration being present in the reaction mixture. The primer mix contained solutions of four primers and comprised 84 µL of TE buffer and 2 µL of each primer (for both forward and reverse for a total of 16 µL of primers).

The PCR products were identified based on their sizes using an Applied Biosystems 3130*xl* sequencer and GeneMapper ver. 4.0 (Applied Biosystems, USA). The fragment analysis of each sample was performed using a reaction mixture of 14.3 µL of formamide, 0.2 µL of Applied Biosystems GeneScan 500LIZ Size Standard (Applied Biosystems), and 0.5 µL of previously amplified DNA.

### Statistical Analysis

The distributions of the metal concentrations in bank vole liver and kidney samples from each population were checked for normality using the Kolmogorov–Smirnov test (using Statistica ver. 9.0 software package [StatSoft, Tulsa, Oklahoma, USA]). Most of the data did not form a normal distribution, so they were log-transformed. We used a two-way analysis of variance (ANOVA) to test the significance of the effects of the sex of the individuals and the site on metal accumulation in the tissues of the animals from different populations. Sex was not a significant factor, so the rest of the analyses were performed on the combined data for both sexes. We used one-way ANOVA and Tukey post hoc test to determine the significance of the differences between the populations and the effects of the population type. We used main-effects ANOVA to study the effects of the geographical region (South versus North populations) and the site (the polluted sites were excluded from this analysis because there were no such sites in northern Poland). These statistical analyses were also performed using Statistica ver. 9.

Data related to the genetic diversity were analyzed using GenAlEX 6.4. We calculated the expected heterozygosity (*H*_e_), the unbiased expected heterozygosity (UH_e_), the observed heterozygosity (*H*_o_), the average number of alleles at each locus (*N*_a_), the number of private alleles, and the fixation index (*F*, calculated as *F* = (*H*_e_ − *H*_o_)/*H*_e_). We performed analysis of molecular variance (AMOVA) based on the *F*_st_ values. FSTAT software (http://www2.unil.ch/popgen/softwares/fstat.htm) was used to assess the allelic richness, the gene diversity (*H*_s_) (Nei 1987), and differences between the population types (island, clean-mainland, and polluted) in terms of allelic richness, i.e., *H*_o_ and *H*_s_.

Phylip 3.69 (Felsentein 2009) was used to generate an UPGMA tree based on Nei’s genetic distance (Nei and Roychoudhury 1974). We used Wilcoxon one-tailed sign-rank test to determine the heterozygosity excess in the Bottleneck software to assess the possibility that bottlenecks had occurred in the populations studied assuming a two-phase mutation model (70 % stepwise mutation model and a 30 % infinite alleles model) (Cornuet and Luikart 1997). Sign-rank test is recommended for analyzing results for no fewer than four loci, and our protocol met this criterion. Bayesian population assignment was performed to infer genetic structure of populations and to assess number of genetic clusters using STRUCTURE software (Falush et al. 2003, 2007; Hubisz et al. 2009; Pritchard et al. 2000). Admixture model with uncorrelated frequencies was used. We ran ten independent runs, for population numbers *K* = 1 to *K* = 10, using a burn-in period of 100,000 iterations, and we collected data for 500,000 iterations. STRUCTURE HARVESTER software was used for collating results generated with STRUCTURE (Earl and vonHoldt 2012). Mantel test was performed using the *F*_*st*_ values and the geographic distances between the populations (Isolation By Distance Web Service, version 3.23) (Jensen et al. 2005).

## Results

### Metal Concentrations

**Fig. 2:**
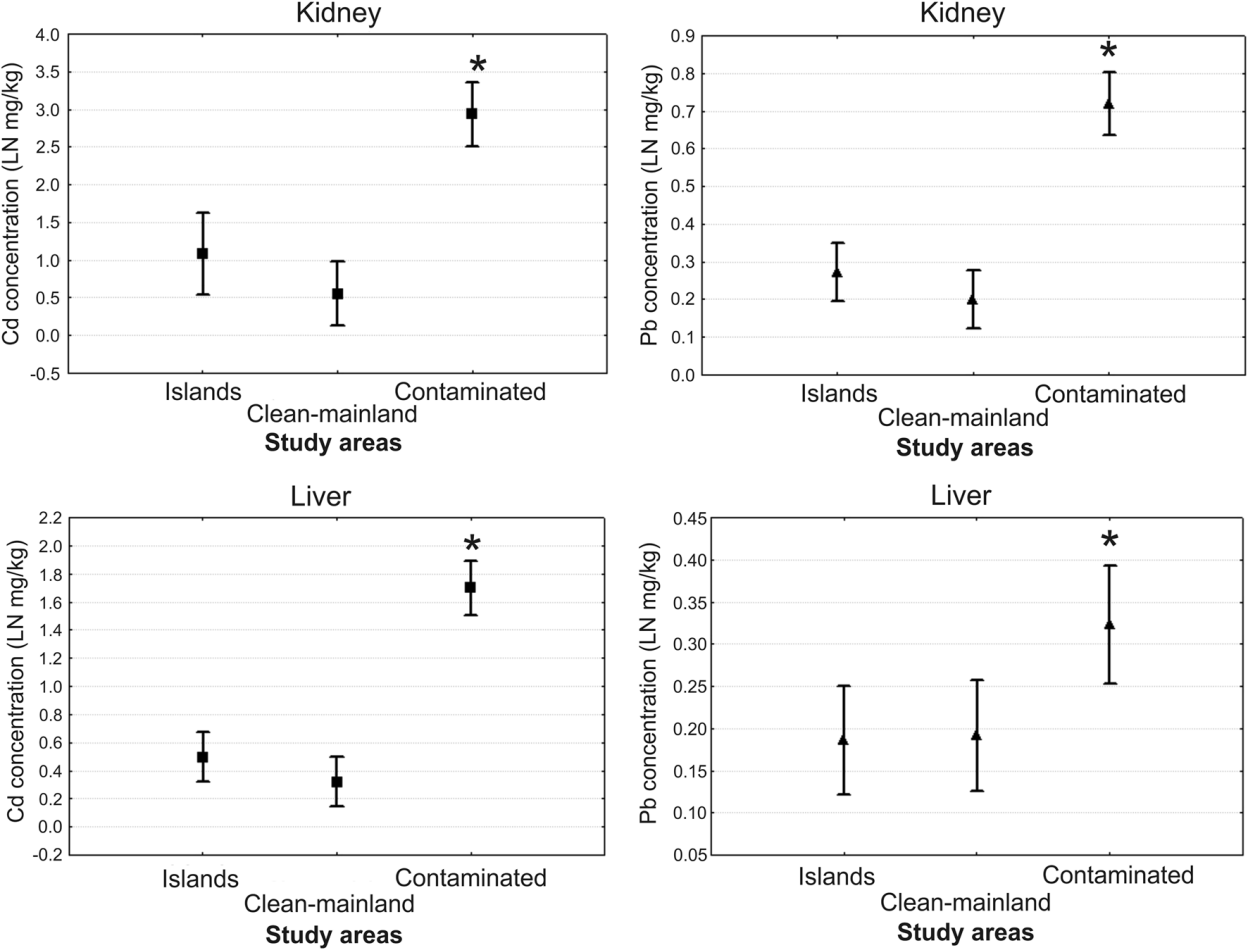
Mean concentrations (±SE) of lead and cadmium in livers and kidneys of bank voles *M. glareolus* from different population types (*statistically significant difference, *p* < 0.05)

The average lead concentration was highest in livers of bank voles from POl (0.77 mg/kg dw) (Table 1). It was ≤3 times greater than average lead concentrations in the livers of animals from the two other polluted sites, PMi (0.35 mg/kg dw) and PKa (0.23 mg/kg dw). Lead concentrations in livers of animals from the island and clean-mainland populations were not much lower ranging from 0.18 to 0.30 mg/kg dw. Cadmium concentrations in liver samples from the populations of the clean and polluted areas were more clearly different (Table 1; Fig. 2). Cadmium concentrations in livers of bank voles from the polluted sites ranged from 5.10 mg/kg dw at POl to 10.43 mg/kg dw at PKa. Cadmium concentrations in livers of the animals from the clean areas (the islands and the mainland sites) were 0.23–2.07 mg/kg dw.

Lead concentrations were generally greater in kidneys than in livers: from 0.74 to 1.32 mg/kg dw in the animals from the polluted sites and from 0.12 to 0.47 mg/kg dw in the animals from the clean sites (Table 1). Cadmium concentrations were particularly high in kidneys of animals from the polluted sites: 40.66 mg/kg dw in the PKa samples, 20.00 mg/kg dw in the PMi samples, and 16.00 mg/kg dw in the POl samples.

**Fig. 3:**
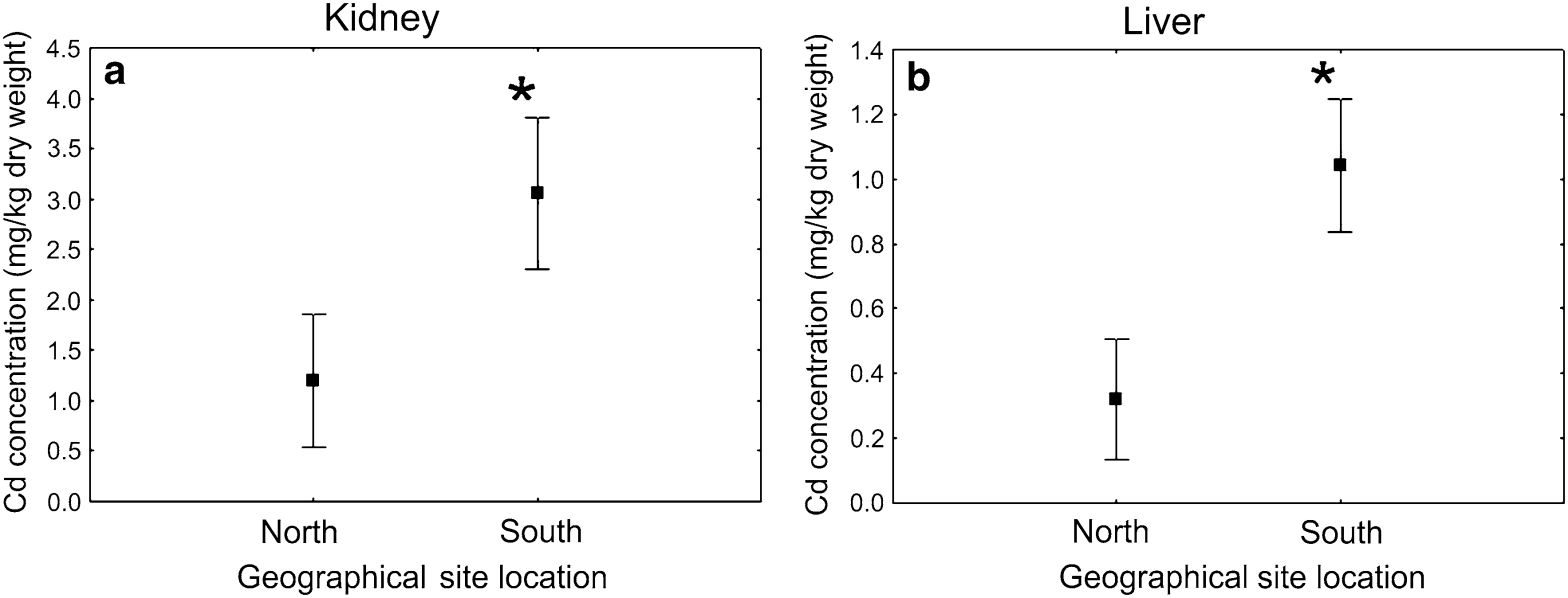
Cadmium concentration (±SE) in kidneys (**a**) and livers (**b**) of *M. glareolus* from northern and southern regions of Poland (polluted populations excluded) (*statistically significant difference, *p* < 0.001)

ANOVA and Tukey test showed that cadmium concentrations in both livers and kidneys did not differ significantly between island and clean-mainland populations, but they differed between polluted and island as well as between polluted and clean-mainland populations (Fig. 2). There was a statistically significant north/south difference in cadmium concentration (excluding populations from the polluted sites) but not in lead concentrations (Fig. 3).

### Genetic Diversity

**Fig. 4:**
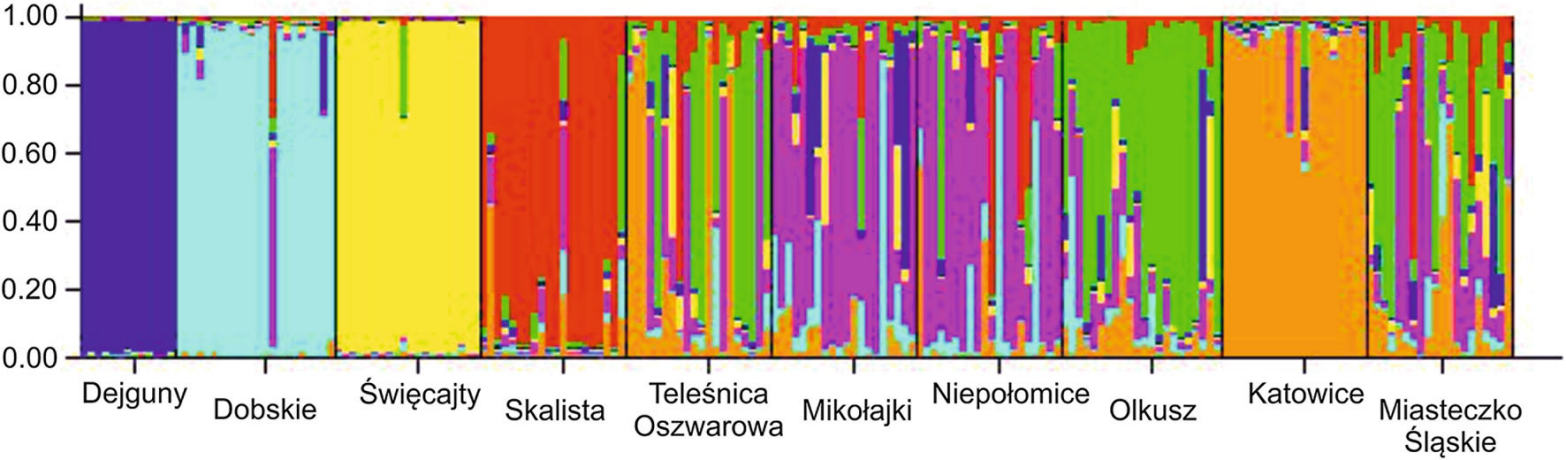
Results of STRUCTURE analysis based on eight microsatellite loci for seven optimal number of clusters

Mean UH_e_ ranged from 0.602 to 0.891 (Table 2). UH_e_ was close to the bottom of this range for the island populations. UH_e_ for the populations from the clean-mainland and the polluted sites, except for PKa, were comparable. The allelic richness for the studied populations varied in a similar way to the expected heterozygosity. The number of alleles was highest for the clean-mainland and polluted populations, except for the PKa population (Table 2). *N*_a_ values were smallest for the IDe and ISw populations. The mean number of private alleles was low, <1, for all of the populations (Table 2). No private alleles were found for the ISw population. The mean number of private alleles for all loci per population was highest (0.875) for the PMi population. The *F* value was negative for two of the island populations, IDe and ISw (Table 2), and ranged between 0.061 and 0.154 for the other populations. AMOVA calculated on the basis of *F*_st_ values (Table 3) indicated that 80 % of the molecular variance was within individuals; 10 % of the variance was among populations; and 10 % of the variance was among individuals. Bayesian population assignment showed the highest probability for existence of seven genetic clusters (Fig. 4) (Evanno et al. 2005) and indicated that water is a sufficient barrier to isolate these small rodents. Very limited gene flow was also shown for the PKa population. Mantel test performed with the *F*_st_ values, and geographic distances showed that there was no correlation between genetic distance and geographic distance (*p* = 0.89).

**Table 2:** Parameters of genetic diversity calculated for bank vole *M. glareolus* populations

Population	*N*	*N*_a_	Allelic richness	*N*_P_	*H*_o_	*H*_e_	UH_e_	*F*
Mikołajki (CMi)	20	10.3	9.031	0.375	0.741	0.844	0.866	0.121
Niepołomice (CNi)	20	10.3	9.164	0.500	0.714	0.845	0.867	0.154
T. Oszwarowa (CTe)	20	11.3	9.973	0.500	0.817	0.869	0.891	0.061
Katowice (PKa)	20	7.9	6.986	0.375	0.704	0.774	0.794	0.097
M. Śląskie (PMi)	20	11.3	9.875	0.875	0.798	0.859	0.882	0.074
Olkusz (POl)	22	10.4	9.151	0.625	0.731	0.854	0.874	0.142
Dejguny (IDe)	13	3.5	3.500	0.500	0.712	0.578	0.602	−0.239
Dobskie (IDo)	22	8.3	7.178	0.250	0.710	0.786	0.805	0.104
Skalista (ISo)	20	8.6	7.857	0.125	0.748	0.806	0.827	0.072
Święcajty (ISw)	20	5.0	4.524	0.000	0.656	0.636	0.653	−0.031

*N*_*a*_ mean number of alleles for all loci, *N*_*P*_ mean number of private alleles, *H*_o_ observed heterozygosity, *H*_*e*_ expected heterozygosity; *UH*_*e*_ unbiased expected heterozygosity, *F* fixation index

**Table 3:** *F*_ST_ values under diagonal, Nei’s genetic distance above diagonal, for studied populations of bank vole *M. glareolus*

Population	*N*	IDe	IDo	ISw	ISo	CTe	CMi	CNi	POl	PKa	PMi
IDe	20	−	1.165	1.621	0.956	1.001	0.955	1.312	0.937	0.980	1.026
IDo	20	0.141	−	1.112	1.123	0.840	0.588	0.637	0.719	1.094	0.855
ISw	20	0.207	0.121	−	1.428	0.818	0.926	1.221	0.875	1.254	0.843
ISo	20	0.126	0.079	0.131	−	0.529	0.675	0.672	0.691	0.897	0.590
CTe	20	0.117	0.057	0.092	0.040	−	0.514	0.535	0.346	0.472	0.282
CMi	22	0.118	0.048	0.101	0.050	0.033	−	0.387	0.640	0.730	0.523
CNi	13	0.134	0.051	0.115	0.049	0.034	0.029	−	0.531	0.739	0.478
POl	22	0.115	0.054	0.097	0.049	0.023	0.040	0.035	−	0.606	0.419
PKa	20	0.132	0.086	0.131	0.073	0.041	0.059	0.058	0.050	−	0.585
PMi	20	0.120	0.059	0.094	0.044	0.019	0.034	0.032	0.028	0.049	−

There were significant differences in *H*_*s*_ and allelic richness values only between the island and clean-mainland populations (Table 4). The Nei genetic distance values suggested that the island populations are distant from the mainland populations (Table 3). Wilcoxon test indicated that there were bottleneck effects in the IDe, CTe, CNi, POl, and PMi populations. The genetic diversity parameters in the loci are listed in Table 5.

**Table 4:** Parameters of genetic variation for different population types generated with FSTAT ver. 2.9.3. (A) two-sided *p* values obtained after 5,000 permutations for comparison of different population types (FSTAT ver. 2.9.3) (B)

(A)				(B)			
Type of population	Allelic richness	*H*_o_	*H*_s_	Comparison model	Allelic richness *p* values	*H*_o_ *p* values	*H*_s_ *p* values
Island	5.765	0.705	0.735	Island vs. Clean-mainland	0.021	0.171	0.028
Clean-mainland	9.389	0.757	0.878	Island vs. Polluted	0.085	0.323	0.090
Polluted	8.670	0.744	0.854	Clean-mainland vs. Polluted	0.715	0.743	0.761

**Table 5:** Parameters of genetic diversity of bank vole *M. glareolus* populations calculated for each studied locus

Study site	Locus							
	LIST305	MSCg20	LIST303	LIST002	MSCg7	MSCg4	MSCg9	MSCg24
IDe								
*N*_a_	2	3	4	5	4	5	2	3
*H*_o_	0.769	0.538	0.308	0.923	0.846	1.000	0.769	0.538
*H*_e_	0.497	0.630	0.544	0.716	0.642	0.713	0.473	0.411
*F*	−0.548	0.146	0.435	−0.289	−0.318	−0.402	−0.625	−0.309
IDo								
*N*_a_	8	10	10	11	8	7	8	4
*H*_o_	0.818	0.818	0.500	0.682	0.727	0.773	0.905	0.455
*H*_e_	0.813	0.845	0.772	0.808	0.796	0.807	0.793	0.658
*F*	−0.006	0.032	0.352	0.156	0.087	0.042	−0.142	0.309
ISw								
*N*_a_	4	6	5	7	6	4	5	3
*H*_o_	0.500	0.700	0.650	0.500	0.850	0.600	0.750	0.700
*H*_e_	0.488	0.736	0.696	0.488	0.751	0.606	0.674	0.651
*F*	−0.026	0.049	0.066	−0.026	−0.131	0.010	−0.113	−0.075
ISo								
*N*_a_	8	10	8	9	7	10	9	8
*H*_o_	0.650	0.500	0.650	0.750	0.850	0.950	0.895	0.737
*H*_e_	0.705	0.829	0.796	0.840	0.789	0.845	0.816	0.825
*F*	0.078	0.397	0.184	0.107	−0.078	−0.124	−0.097	0.107
CTe								
*N*_a_	10	9	12	12	13	12	10	12
*H*_o_	0.900	0.400	0.900	0.550	0.947	0.947	0.895	1.000
*H*_e_	0.826	0.830	0.888	0.883	0.902	0.882	0.864	0.874
*F*	−0.089	0.518	−0.014	0.377	−0.051	−0.074	−0.035	−0.144
CMi								
*N*_a_	9	13	9	9	9	10	13	10
*H*_o_	0.950	0.450	0.600	0.526	0.850	0.850	0.800	0.900
*H*_e_	0.853	0.851	0.869	0.823	0.798	0.866	0.850	0.843
*F*	−0.114	0.471	0.309	0.360	−0.066	0.019	0.059	−0.068
CNi								
*N*_a_	7	9	13	11	13	9	10	10
*H*_o_	0.750	0.421	0.750	0.389	0.950	0.750	0.800	0.900
*H*_e_	0.789	0.867	0.874	0.844	0.906	0.808	0.811	0.860
*F*	0.049	0.514	0.142	0.539	−0.048	0.071	0.014	−0.047
POl								
*N*_a_	8	12	11	12	9	12	9	10
*H*_o_	0.773	0.455	0.864	0.636	0.682	0.818	0.714	0.909
*H*_e_	0.799	0.896	0.886	0.877	0.810	0.883	0.814	0.871
*F*	0.032	0.493	0.026	0.274	0.158	0.074	0.123	−0.044
PKa								
*N*_a_	7	6	11	7	7	9	9	7
*H*_*o*_	0.850	0.450	0.700	0.579	0.650	0.750	0.900	0.750
*H*_e_	0.809	0.691	0.808	0.744	0.763	0.789	0.853	0.739
*F*	−0.051	0.349	0.133	0.222	0.148	0.049	−0.056	−0.015
PMi								
*N*_a_	10	8	13	11	10	11	15	12
*H*_o_	0.850	0.550	0.700	0.684	0.800	0.900	0.900	1.000
*H*_e_	0.838	0.805	0.890	0.837	0.850	0.880	0.904	0.873
*F*	−0.015	0.317	0.213	0.182	0.059	−0.023	0.004	−0.146

*N*_*a*_ mean number of alleles per loci, *H*_*o*_ observed heterozygosity, *H*_*e*_ expected heterozygosity, *F* fixation index

## Discussion

Human activities have dramatically altered the natural environment. As a result, organisms must adapt so that they can survive pollution, habitat fragmentation, climate change, and other changes. Factors that can disturb the genetic stability of a population are those that act directly on the genome (by changing the DNA integrity, causing mutations, and affecting the DNA repair system); other factors act indirectly by decreasing the population size. Contamination and isolation can both affect the genome in two ways, either decreasing or increasing genetic diversity. Biomarkers, such as microsatellites, have high mutation rates and high degrees of variability, and they can be used to determine changes in genetic diversity (Mussali-Galante et al. 2014). However, mortality is driven by natural selection if it is dependent on fitness rather than being random (De Wolf et al. 2004). In this case, a toxicant will act as a selective agent on certain loci, at which point genetic diversity will decrease, but the genetic diversity in general will not be affected. It will not be possible to use microsatellites or other neutral biomarkers to obtain complete knowledge of the changes in genetic diversity in such particular cases. However, the role of transcriptomial regulation in stress response adaptation has been emphasized in recent studies (Roelofs et al. 2010).

Our attempt to assess the relationship between genetic diversity and metal pollution or isolation was performed to follow up the conflicting results of previous research (Berckmoes et al. 2005; Ungherese et al. 2010) and because of our poor understanding of genetic variation in terrestrial rodents.

### Metal Pollution

The populations that were under pressure from pollution were exposed mainly to metals, particularly cadmium, lead, and zinc, from past and/or current industrial activity. Exposure to the pollutants was chronic, but the metals that are toxic to rodents, cadmium and lead, did not reach high levels in the tissues of the animals that were collected (Table 1). The types and levels of contamination found in this study can be found in a number of places where mining and processing of zinc and lead ores occur (Dejonghe 1998; John et al. 1975; Milton et al. 2003; Stafilov et al. 2010). The results of this study of pollution and genetic variation may be used to predict potential genetic changes in populations inhabiting similarly contaminated environments.

Lead and cadmium concentrations were significantly greater in livers and kidneys of bank voles from the polluted sites, PMi, PKa, and POl, than in those from individuals from the islands and the clean-mainland areas (Table 1; Fig. 2). Moreover, cadmium concentrations in the bank vole tissues were greater (≤40 mg/kg dw in kidneys from the PKa population) than lead concentrations in all populations. In general, livers accumulated less of each metal than kidneys, indicating that the voles were intensively accumulating and excreting metals through the kidneys. Cadmium levels in bank vole livers differed more clearly between populations, and the statistical test separated the polluted sites from the clean sites (island and mainland) (Fig. 2).

The lead concentration in kidneys of bank voles from the most polluted site (1.32 mg/kg dw) was several times greater than those of individuals from the clean sites in this study and in other studies: 0.3 mg/kg dw (Milton et al. 2003) and 0.74 mg/kg dw (Damek-Poprawa and Sawicka-Kapusta 2004). Particularly high level of cadmium noted in the vicinity of a nonferrous smelter at PKa (already closed) provides clear evidence that metal pollution is persistent and can affect living organisms even if the contaminants were last released several years earlier.

### Effect of Isolation on Genetic Variation

Our results confirmed that the water barrier and small population size in island populations may contribute to a decrease in genetic diversity. UH_e_ was lower for island populations of IDe and ISw than for the other populations, suggesting that there had been a decrease in diversity in these populations. Allelic richness in all populations (Table 2) followed a similar pattern to that seen with UH_e_. Bayesian genetic clustering identified limited gene flow between the mainland and island populations as well as the PKa population (Fig. 4). This shows that the PKa population is probably isolated, which is consistent with the fact that the forest inhabited by the bank vole population is surrounded by roads and industry. The region of Silesia is particularly affected by humans, and coal and ore mining and metal smelting have been common for many years. Differences between island populations in genetic variation parameters are a consequence of their isolation and their history. The ISo population, which had the highest level of diversity among the island populations, is the youngest one, having been created artificially by animals from the nearby mainland being brought to the island in 2005 (Boratyński and Koteja 2009, 2010). As is clear from other studies, water is a difficult barrier for *M. glareolus* to cross. Kozakiewicz et al. (2009) found greater differences between island and mainland populations than between two mainland populations inhabiting different sites and noted that *M. glareolus* was more vulnerable to this type of isolation than was *Apodemus flavicollis*. Mantel test did not show a correlation between genetic distance and geographic distance in the study presented here, and this also suggests that factors such as isolation may increase genetic distance.

### Effect of Pollution on Genetic Variation

We analyzed the microsatellite DNA of our bank vole populations and found that exposure to a contaminated environment had no effect on genetic variation, as expressed as the average number of alleles per locus or as heterozygosity, in *M. glareolus* populations. Only the island and clean-mainland populations differed significantly in terms of gene diversity (*H*_s_) and allelic richness (Table 4). The populations of the polluted sites (PKa, POl, and PMi) did not differ significantly from the populations of the clean-mainland sites (CNi, CMi, and CTe) in terms of the number of alleles or expected and observed heterozygosity (Tables 2 and 4). Comparisons between individual populations showed that the values for genetic parameters (especially *N*_a_) were slightly lower only for the PKa population than for the other mainland populations, including those from polluted areas. This may be explained in two ways. Metals may affect genetic diversity (1) by causing a decrease in effective population size and leading to a bottleneck effect or (2) by selection (Van Straalen and Timmermans 2002). The PKa population has been exposed to metal contamination for generations, longer than the populations of the other polluted sites have been exposed, and this was confirmed by tissue loads of cadmium being greater in the PKa population than in any of the others (Table 1). However, the location and size of the site may have played a significant role. The PKa population is located between large motorways and a highway, and its habitat is substantially smaller than the those of populations at the POl and PMi sites. These factors have caused this population to become almost as isolated as it would be on an island, and this is consistent with the Bayesian analysis results (Fig. 4). Previous research into bank voles (Gerlach and Musolf 2000; Redeker et al. 2006) showed that the presence of a highway may severely decrease bank vole migration.

Genetic diversity parameters for the populations of the other two polluted sites, POl and PMi, did not appear to be different from the those for the clean-mainland populations (Table 2). One explanation for this might be that gene flow obviated the negative consequences of metal pollution. Both the POl and PMi populations are located in large forests. Similar results were reported for the rodent *Dipodomys merriami* exposed to radionuclides (Theodorakis et al. 2001). In another example, *Sigmodon hispidus* showed no differences in genetic diversity between populations of clean sites and those of oil refinery sites (Pfau et al. 2001). The investigators of those studies suggested that migration tempered the effects of selection. A second explanation is that because cadmium (a potential inducer of mutation) concentrations in tissues of bank voles from the POl and PMi populations were half those in the tissues from the PKa population, the effect of cadmium was presumably weaker at those two sites than at the PKa site.

The lack of difference in *H*_*s*_ between the island and the polluted site populations [the effect of population type (Table 4B)] could be the result of different processes leading to similar consequences. Whether or not genotoxic effects of metal pollution can be confirmed, indirect effects (decreasing the effective population size) cannot be ruled out. High inbreeding coefficients for *M. glareolus* populations living close to a copper smelter in the Ural Mountains was found to be the result of decreased populations (Gileva et al. 2008). Berckmoes et al. (2005), in contrast, found high levels of genetic diversity in the populations from both clean and contaminated sites.

## Conclusion

Genetic diversity parameters showed that island populations had low levels of genetic variation, confirming that water is an effective barrier for bank vole populations. Nei’s genetic distance results (Table 3) and Bayesian clustering results (Fig. 4) confirmed that the island populations are distant not only from each other but also distant from the other populations. Isolation also probably influenced one of the polluted sites, PKa, the population of which had lower levels of heterozygosity that the other mainland populations had. The lack of distinct pollution effect on genetic diversity of the two remaining polluted populations, POl and PMi, may have been the result of the mild genotoxicity of pollutants at those sites and the migration ability of this small rodent species (Petrusewicz 1983), both of which have increased the level of genetic variability at these sites.
